# Urban Rail Transportation and SARS-Cov-2 Infections: An Ecological Study in the Lisbon Metropolitan Area

**DOI:** 10.3389/fpubh.2021.611565

**Published:** 2021-02-03

**Authors:** Milton Severo, Ana Isabel Ribeiro, Raquel Lucas, Teresa Leão, Henrique Barros

**Affiliations:** ^1^EPIUnit - Instituto de Saúde Pública, Universidade do Porto, Porto, Portugal; ^2^Departamento de Ciências da Saúde Pública e Forenses e Educação Médica, Faculdade de Medicina, Universidade do Porto, Porto, Portugal

**Keywords:** SARS-CoV-2, transport policy, disease transmission, coronavirus (2019-nCoV), urban health

## Abstract

**Introduction:** The large number of passengers, limited space and shared surfaces can transform public transportation into a hub of epidemic spread. This study was conducted to investigate whether proximity to railway stations, a proxy for utilization, was associated with higher rates of SARS-CoV-2 infection across small-areas of the Lisbon Metropolitan Area (Portugal).

**Methods:** The number of SARS-CoV-2 confirmed infections from March 2 until July 5, 2020 at the parish-level was obtained from the National Epidemiological Surveillance System. A Geographic Information System was used to estimate proximity to railway stations of the six railway lines operating in the area. A quasi-*Poisson* generalized linear regression model was fitted to estimate the relative risks (RR) and corresponding 95% confidence intervals (95%CI).

**Results:** Between May 2 and July 5, 2020, there were a total of 17,168 SARS-CoV-2 infections in the Lisbon Metropolitan Area, with wide disparities between parishes. Overall, parishes near any of the railway stations of the *Sintra* line presented significantly higher SARS-CoV-2 infection rates (*RR* = 1.42, 95%CI 1.16, 1.75) compared to parishes located farther away from railway stations, while the opposite was observed for parishes near other railway stations (*Sado* and *Fertagus* lines), where infection rates were significantly lower than those observed in parishes located farther away from railway stations (*RR* = 0.66, 95%CI 0.50, 0.87). The associations varied according to the stage of the epidemic and to the mitigation measures enforced. Regression results also revealed an increasing influence of socioeconomic deprivation on SARS-CoV-2 infections.

**Conclusions:** No consistent association between proximity to railway stations and SARS-CoV-2 infection rates in the most affected metropolitan area of Portugal was observed, suggesting that other factors (e.g., socioeconomic deprivation) may play a more prominent role in the epidemic dynamics.

## Introduction

By the beginning of 2020, societies worldwide were experiencing an unprecedented, disruptive event—the coronavirus disease 2019 (COVID-19) pandemic caused by the severe acute respiratory syndrome coronavirus 2 (SARS-CoV-2). The first confirmed case of COVID-19 in Europe was identified on January 23, 2020, and in Portugal, the first case was diagnosed on March 2, 2020 (1). Considering the most recent data available (from September 14, 2020), there were a total of 65,021 confirmed cases of COVID-19 and 1,875 deaths in Portugal.

Urban areas have been epicenters of the COVID-19 pandemic (2). In Portugal, reported SARS-CoV-2 infections are concentrated in the two main metropolitan areas of the country: the Lisbon Metropolitan Area and the Porto Metropolitan Area, which account for 46 and 23% of all reported infections, respectively, while concentrating 28 and 17% of the population in the country. Many individuals who live, work and attend school in urban areas use public transportation (metro, bus and trains) for their daily commute. Public transportation may act as a hub for epidemic spread, since there is a high number of individuals near each other in closed spaces, making it difficult to keep a safe distance (3). The existence of various surfaces, such as seats, handrails, doors, and ticket machines, shared daily by thousands of individuals, also facilitate the transfer of the SARS-CoV-2 virus (4), which is believed to persist on surfaces for several hours (5). Notably, public transportation is used more frequently by low-income individuals, who are unable to opt for other modes of transportation, such as a personal car (6). Additionally, recent studies suggest that low-income individuals are more likely to be affected by most of the known risk factors for COVID-19, such as poor housing conditions, the presence of comorbidities and providing essential services, including industry, cleaning, food supply or construction (7–9).

During the lockdown period, the role of public transportation on epidemic spread was not considered particularly relevant as most countries recorded a drastic reduction in the use of public transportation (10). In Portugal, the State of Emergency was declared on March 19, 2020 and was renewed biweekly until May 2, 2020 (11). The State of Emergency measures enforced the closure of international borders, and the suspension of non-essential services and events. Residents could only leave their homes to shop for basic needs, to take care of vulnerable individuals, to walk their dogs or dispose of daily residuals and to go to work. Traveling to work was limited to those in essential services and working from home was encouraged. Consequently, between March and May 2020, a remarkable decrease of about 75% in the utilization of public transportation was observed in Portugal (10).

As the lockdown eased, and workplaces and services reopened, the use of public transportation increased, although as of September 2020, it still remained below pre-lockdown levels (12). The Lisbon Metropolitan Area is the most populated metropolitan area of the country and concentrates the highest proportion of residents that use public transportation on a daily basis, namely urban trains. According to the most recent Population and Housing Census (2011), 7.6% of the population from the Lisbon Metropolitan Area uses trains as their main mode of transportation vs. 2.9% in the entire country (13).

Coincidentally, after the lockdown was lifted, from June onwards, the Lisbon Metropolitan Area recorded the greatest growth in the number of SARS-CoV-2 infections, and in July, three quarters of all daily cases reported in Portugal were among residents of the Lisbon Metropolitan Area. Lay media and other stakeholders began to question whether the upsurge in SARS-CoV-2 infections in the Lisbon Metropolitan Area could be related with the large number of passengers that use the Lisbon Urban railway services on a daily basis for their commute.

However, national evidence on this potential association is absent and international evidence on the topic is very limited. As far as we are aware, there is one single comprehensive study on the community exposures associated with SARS-CoV-2 infection (14). In this study, authors found that the use of public transportation was not significantly associated with SARS-CoV-2 infection in the USA. However, the authors highlight that the study participants may not be representative of the universe and that the sample size may be insufficient. Moreover, the studies focused on the association between rail transportation and SARS-CoV-2 are from China (15, 16), whose epidemiological situation and disease control measures are completely different from those in place in European countries, such as Portugal, making it difficult to transpose their findings to the European/Portuguese context. A study conducted in China examined the factors influencing the number of imported cases from Wuhan as well as the spread speed and pattern of the pandemic, and found that the presence of an airport or high-speed railway station was associated with the speed of the infection spread, although its link with the number of confirmed cases was weak (15). Another study, also focused on Wuhan, reported a strong association between travel by train to six major Chinese cities and the number of SARS-CoV-2 cases (16). However, these studies did not evaluate the role of short-duration urban train trips, typically used for daily commuting, on the number of SARS-CoV-2 infections. Additionally, little is known about the role of public transportation on transmission following the introduction of widespread containment or mitigation measures.

Therefore, this ecological study was conducted to investigate whether proximity to urban railway stations, a proxy for use in daily commuting, was associated with higher rates of SARS-CoV-2 infection across small-areas of the Lisbon Metropolitan Area, between March and July 2020.

## Materials and Methods

### Study Design

This ecological study used parishes (smallest administrative territorial unit) of the Lisbon Metropolitan Area as observation units and compared the SARS-CoV-2 infection rates between parishes closer to and parishes farther away from a train station, operationally defined below, while considering the specific train line that passes the closest station.

### Study Area

The Lisbon Metropolitan Area NUT III (Nomenclature of Territorial Units for Statistics III) is in the center-south region of Portugal, and includes 18 municipalities and 118 parishes. It is the most populated metropolitan area of the country and, according to the most recent population estimates (31 December 2018), has 2.86 million inhabitants. It should be noted that, although the geographical overlap is not comprehensive, the Lisbon Metropolitan Area includes 78% of the population from the Lisbon Health Administration Region (*Administração Regional de Saúde de Lisboa e Vale do Tejo*, ARSLVT), to which reported infection cases are assigned.

### SARS-CoV-2 Infections

Data on the cumulative number of SARS-CoV-2 infections in the Lisbon Metropolitan Area, according to the parish of occurrence, from March 2 until July 5, 2020 were used. Data were obtained from the National Epidemiological Surveillance System (SINAVE) and provided by the Directorate-General of Health (*Direção-Geral da Saúde*, DGS). SINAVE is a real-time electronic platform used by public, private and social healthcare institutions in Portugal to collect data on communicable diseases and other public health risks (17). The information collected is based on international standards for disease surveillance, recommended by the European Center for Disease Prevention and Control, and the World Health Organization, and reported on the electronic form provided by SINAVE on https://sinave.min-saude.pt (17). Following the submission of the notification by an authorized user, data are available in real-time for the local, regional and national health authority. Additionally, data are consecutively validated, by hierarchical level, to ensure the validity of the reported information and to avoid duplicate cases.

The dataset was provided by DGS at the individual-level—each line representing a SARS-CoV-2 infection and the associated variables, namely the parish of occurrence, from which, case counts according to the parish were computed.

### Train Station Network and Geospatial Procedures

The list of railway stations in the Lisbon Metropolitan Area and the corresponding railway lines were obtained from the CP—Comboios de Portugal (https://www.cp.pt/passageiros/pt) and Fertagus (https://www.fertagus.pt/) websites. The location (geographical coordinates) of each railway station was collected using Google Maps and converted into a point map using a Geographic Information System (ArcGIS 10.7.1).

**Figure 1:**
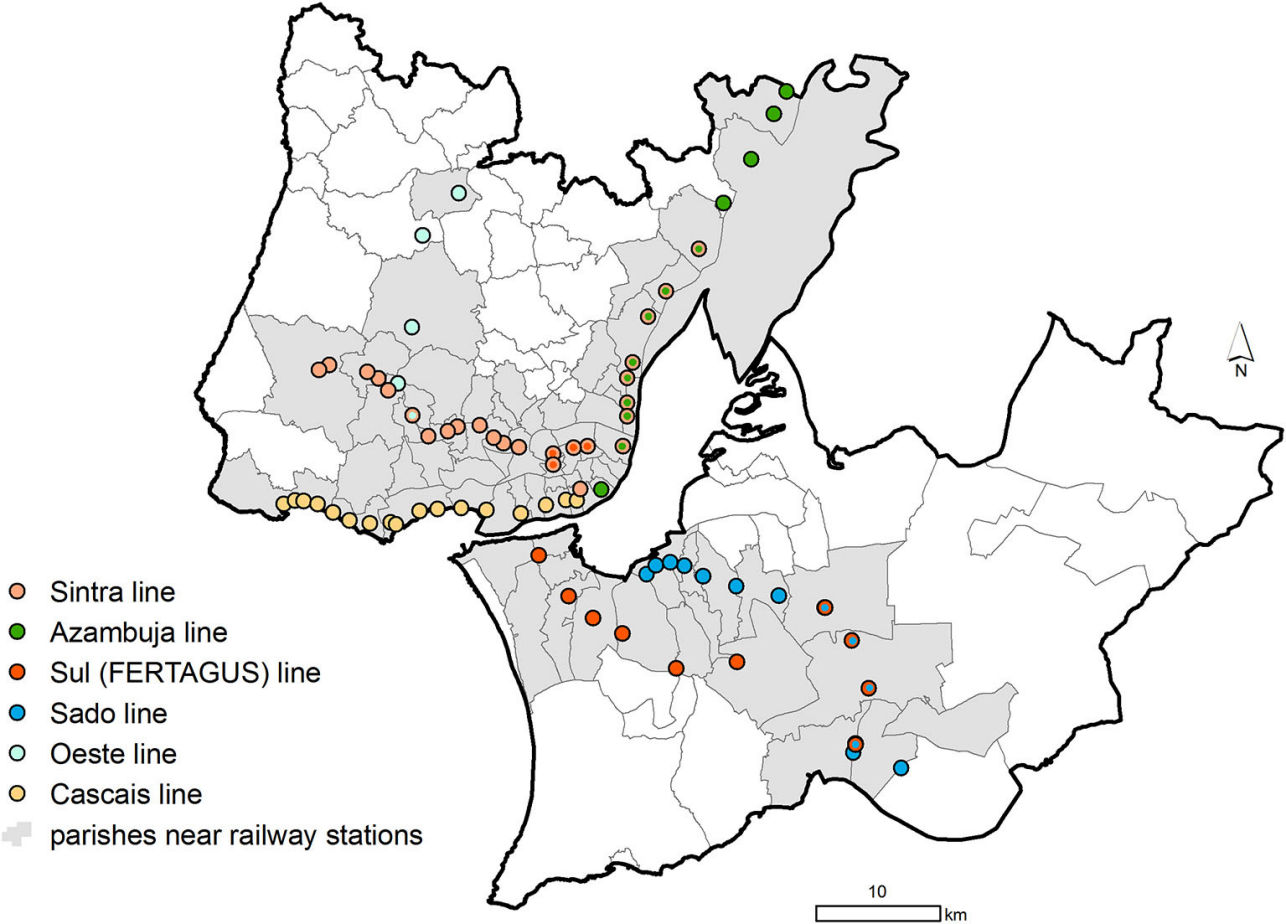
Location of the railway stations from the Lisbon Metropolitan Area in 5 July 2020.

There were a total of six railway lines operating in the study area when this study was conducted (7 July 2020). Four railway lines belonged to *Comboios Urbanos de Lisboa* (*Linha de Azambuja, Linha do Sado, Linha de Sintra*, and *Linha de Cascais*) and two lines belonged to *Comboios Regionais/Suburbanos* (*Linha do Oeste* and *Linha do Sul-Fertagus*), including a total of 71 railway stations depicted in Figure 1.

Using the official administrative cartography as a base map [*Carta Administrativa Oficial de Portugal* version 2019, CAOP 2019 (18)] and a Geographic Information System (ArcGIS 10.7.1), a 100 × 100 meter grid covering all of the parishes belonging to the Lisbon Metropolitan Area was created. Afterwards, using the centroid of each grid cell as the origin, the Euclidian distance (i.e., straight line) from that centroid to the nearest railway station was computed. The shortest distance from each parish to the nearest railway station was computed with the resultant distance matrix. Because each parish can be close to various stations, served by different railway lines, each parish was assigned to the line that ran through the nearest station. Then, the parishes were grouped into: parishes nearer railway stations (those at a mean minimum distance equal to or shorter than 3,000 meters, *n* = 76) and parishes farther away from railway stations (those at a mean minimum distance larger than 3,000 meters, *n* = 42). The parishes classified as near railway stations are depicted in gray in Figure 1.

### Statistical Analysis

To estimate the magnitude of the association between SARS-CoV-2 infection rates and proximity to a railway station at the parish-level (nearer vs. farther), a quasi-*Poisson* generalized linear regression model with the log of the population [obtained from Statistics Portugal (19)] as the offset was fitted to estimate the relative risks (RR) and corresponding 95% confidence intervals (95%CI). An RR was considered statistically significant whenever its 95% CI did not include the value 1. The quasi-*Poisson* generalized linear regression model was used to account for over-dispersion in the data.

First, we applied a quasi-*Poisson* generalized linear regression model to estimate the unadjusted RR for the variables proximity to railway station and time period. Four periods were considered, each representing a stage in the epidemic spread and mitigation measures: March–July (the entire period of analysis, 2 March−5 July), March (the early stage of the epidemic and transition to the State of Emergency, 2–31 March), April (State of Emergency, 1–30 April), May (end of the State of Emergency and beginning of the State of Alert, 1–31 May), and June–July (State of Alert, 1 June−5 July).

Second, an interaction between proximity to railway station and time period was added to the model. Finally, the interaction was adjusted for potential confounding variables. To control for confounding, models were adjusted for socioeconomic deprivation, which was assessed using the European Deprivation Index. The European Deprivation Index was constructed in three steps (20) and resulted from the weighted sum of the following standardized variables at the parish-level: percentage of non-owned households; households without indoor flushing toilet; households with five rooms or less; individuals with blue-collar (manual) occupations; individuals with low educational level (≤6th grade); non-employers; unemployed individuals looking for a job; and foreign residents.

Because there was a statistically significant interaction between proximity to a railway station and the different study periods (*p*-value for interaction < 0.001, obtained from the comparison of the negative binomial generalized linear model with and without the interaction terms), the analysis was stratified according to four periods, each representing a stage in the epidemic spread and mitigation measures.

To check the reliability of our estimates and CI, the quasi-*Poisson* generalized linear regression was compared with the negative binomial generalized linear regression (which can also be used with over dispersed data), and a non-parametric bootstrap approach was performed with 1,000 replicates to assess if the coefficients and the respective variance for the main terms and interaction terms changed (showing similar results). The resampling was performed at the individual-level. From that resampling, cases were counted according to the parish of occurrence. The residuals vs. the fitted plot, the scale-location plot and Cook's distance/leverage plot were used to assess the bias, the heteroscedasticity and the outliers of the models, respectively.

Statistical analysis was performed using R software version 4.0.0. R software and R language are currently the most widely used for statistical analysis of epidemiological data (21).

## Results

Between March 2 and July 5, 2020 there was a total of 17,168 SARS-CoV-2 infections in the Lisbon Metropolitan Area: 1,272 in March, 3,257 in April, 4,566 in May, and 8,073 in June/July. Infection rates across the Lisbon Metropolitan Area varied substantially ranging from 26 cases per 100,000 inhabitants in *Santo Isidoro* (*Mafra*) to 1,725 cases per 100,000 in *Alvalade* (Lisbon) during the entire study period. Table 1 presents the RR comparing infection rates in the parishes near railway stations, according to railway line, with those from parishes located farther away.

**Table 1:** Associations (Relative Risk, RR, and 95% Confidence Intervals, 95%CI) between proximity to railway stations and SARS-CoV-2 infection rates.

	**March–July**	**March**	**April**	**May**	**June–July**
Parishes farther away from railway stations	Ref (1.00)	Ref (1.00)	Ref (1.00)	Ref (1.00)	Ref (1.00)
Parishes near railway stations of the *Azambuja* line	1.15 (0.90, 1.49)	1.26 (0.77, 2.04)	1.11 (0.74, 1.65)	1.19 (0.86, 1.65)	1.06 (0.80, 1.40)
Parishes near railway stations of the *Cascais* line	0.99 (0.75, 1.29)	**1.60 (1.03, 2.51)**	1.14 (0.76, 1.69)	0.77 (0.52, 1.13)	0.89 (0.65, 1.20)
Parishes near railway stations of the *Oeste* line	1.12 (0.68, 1.75)	0.80 (0.24, 2.01)	0.88 (0.36, 1.83)	1.16 (0.60, 2.06)	1.18 (0.69, 1.89)
Parishes near railway stations of the *Sado* and *Fertagus* lines	**0.66 (0.50, 0.87)**	0.72 (0.43, 1.18)	**0.65 (0.42, 0.98)**	0.71 (0.50, 1.01)	**0.61 (0.45, 0.82)**
Parishes near railway stations of the *Sintra* line	**1.42 (1.16, 1.75)**	1.41 (0.95, 2.12)	**1.44 (1.05, 2.01)**	1.27 (0.96, 1.68)	**1.41 (1.13, 1.78)**
Socioeconomic deprivation (European Deprivation Index)	**1.08 (1.05, 1.11)**	0.97 (0.92, 1.01)	**1.05 (1.01, 1.09)**	**1.10 (1.07, 1.14)**	**1.10 (1.07-1.12)**

*Significant associations are bolded*.

Globally, parishes near train stations of the *Sintra* railway line presented significantly higher SARS-CoV-2 infection rates (*RR* = 1.42, 95%CI 1.16, 1.75) compared to parishes located farther away from railway stations, while the opposite was observed for parishes near train stations of the *Sado* and *Fertagus* lines, where infection rates were significantly lower than those in parishes located farther away from railway stations (*RR* = 0.66, 95%CI 0.50, 0.87). However, this pattern changed through the course of the epidemic and according to the measures in place at each time.

In the earlier stage of the epidemic, during March, parishes located near train stations of the *Cascais* railway line presented significantly higher infection rates than those located farther away (*RR* = 1.60, 95%CI 1.03, 2.51). In April, when the entire country was under a lockdown, compared to parishes farther away from railway stations, parishes near railway stations of the *Sado* and *Fertagus* lines presented significantly lower infection rates (*RR* = 0.65, 95%CI 0.42, 0.98), while parishes located near the *Sintra* line presented significantly higher infection rates (*RR* = 1.44, 95%CI 1.05, 2.01). During May, when lockdown measures began to be lifted, no significant associations between proximity to railway stations and SARS-CoV-2 infection rates were observed. During June/July, as in April, parishes near railway stations of the *Sado* and *Fertagus* lines presented significantly lower infection rates (*RR* = 0.61, 95%CI 0.45, 0.82), while parishes located near the *Sintra* line had significantly higher infection rates (*RR* = 1.41, 95%CI 1.13, 1.78).

Regression results also revealed an increasing influence of socioeconomic deprivation on SARS-CoV-2 infection rates. In March, socioeconomic deprivation was not significantly associated with infection rates in parishes, while in April and in May, a positive association between socioeconomic deprivation and SARS-CoV-2 infection rates was observed (April: *RR* = 1.05, 95%CI 1.01, 1.09; May: *RR* = 1.10, 95%CI 1.07, 1.14).

## Discussion

Our results showed an inconsistent association between railway station proximity and SARS-CoV-2 infection rates, as both negative and positive associations were observed depending on the railway line and the time period considered. We found that the most at risk areas changed throughout the epidemic and we observed a time-dependent effect of socioeconomic deprivation on area-level SARS-CoV-2 infection rates.

During April, June/July and globally throughout the COVID-19 epidemic period, we observed that parishes near railway stations of the *Sintra* line had significantly higher SARS-CoV-2 infection rates than parishes farther away from railway stations. The *Sintra* line is one of the most used railway lines of the Lisbon Metropolitan Area ensuring the daily commute of the population from *Sintra* and *Amadora* (the latter is the most densely populated municipality of the country) to the Lisbon municipality (the capital). The *Sintra* line is currently the busiest in the country (and one of the busiest suburban lines in Europe), carrying several tens of thousands of passengers every day with around 14 trains per hour. During the COVID-19 epidemic, physical distancing was strongly encouraged by health authorities, meaning people should stay about 2 m or more away from others. However, trains, such as those in the *Sintra* line, tend to run at maximum capacity during peak hours, making it difficult to implement physical distancing. However, simultaneously, parishes near railway stations of the *Sado* and Fertagus *lines* presented significantly lower SARS-CoV-2 infection rates than those farther away from train stations. The *Sado* and *Fertagus* lines are very busy as well, since they connect the southern parishes of the Lisbon Metropolitan Area located on the left side of the Tagus river estuary to the capital, and carry several tens of thousands of passengers on a daily basis.

Despite this overall pattern, during the earlier stages of the epidemic, the most affected parishes were those crossed by the *Cascais* line. In the beginning of the epidemic in Portugal, most reported cases were imported and associated with national individuals returning from international events (e.g., Milan fashion fairs), Carnival and snow resort holidays, many of them located in Northern Italy. The *Cascais* municipality, despite being socioeconomically heterogeneous, is one of the wealthiest municipalities in Portugal (22) and an important tourist destination (23), which may explain why it constituted a high-rate area during the earlier stages of the epidemic.

Our findings appear to exclude a direct and consistent association between proximity to railway stations and SARS-CoV-2 infections. However, the lack of association observed between variables on an aggregate level do not necessarily represent risk at an individual level. Therefore, we cannot exclude the possibility that some individuals may have acquired the infection inside the train or while at railway stations. Hence, reasonable distancing between passengers should be ensured, face mask usage should be promoted, and the cleaning and disinfecting of surfaces (seats, handrails, doors, and ticket machines) should be strengthened to inactivate the virus (24). This is the first study on this topic in Europe and the first international study exploring the role of intra-urban, short-duration, railway transportation on SARS-CoV-2 infection rates. Thus, it is difficult to establish comparisons with the published literature. The evidence available thus far is from Chinese studies, where airport or high-speed railway station travel was found to be associated to the speed of the infection spread (15). In particular, a strong association between travel by train to six major Chinese cities and the number of SARS-CoV-2 cases has been found (16); and an important SARS-CoV-2 outbreak in public transportation (bus) has also been reported (25).

Though this was not the main objective of the current study, we also observed a consistent increase in the influence of socioeconomic deprivation throughout the epidemic period and after adjusting for the vicinity of railway stations. The existence of socioeconomic inequalities in the risk of SARS-CoV-2 has not been reported in Portugal before and, the widening of socioeconomic inequalities throughout the course of the epidemic, as far as we are aware, has not been described in the national or international literature so far. In the initial stage of the epidemic, no differences in infection rates according to socioeconomic deprivation were observed while, from April onwards, we observed a roughly 10% increase in SARS-CoV-2 infection rates per unit increase in the socioeconomic deprivation index. Socioeconomic and ethnic inequalities in COVID-19 distribution and mortality have been reported in the UK (26), Brazil (27), and the USA (28). Economically disadvantaged individuals are more likely to live in overcrowded houses, a risk factor for respiratory infections (29); to have unstable working conditions and incomes, being more affected by the economic recession caused by the epidemic (29); to have comorbid conditions, which may hamper the immune system's ability to combat the infection (29); and to have lower access to healthcare (30). Additionally, disadvantaged individuals tend to be employed in occupations that do not provide opportunities to work from home during lockdowns (29). This is a very plausible set of explanations for the increase in the socioeconomic inequalities in SARS-CoV-2 infection rates that we observed from April onwards.

This study has limitations that need to be considered. First, the data and analyses were derived from an ecological approach due to the lack of information about individual use of railway transportation, which can be viewed as a weaker approach to causal inference. This is largely accepted at the individual level but ecological causal inference is being increasingly recognized as a major approach to deal with populations as the subject of research.

Also, due to the lack of data on the individual use of railway transportation, we used geographical proximity to railway stations as a proxy of population utilization. The use of proxies and surrogates is a legitimate and common practice in epidemiological research (31, 32), whenever objective data on the exposure and/or outcome of interest is not available, which is the case. On the other hand, in environmental and spatial epidemiology, geographic proximity to emission sources (e.g., major roads, industries) is commonly used as a proxy measure of personal exposure to air pollutants and\noise (33, 34), under the assumption that individuals tend to spend their day within the home surroundings. Accordingly, it seems to us legitimate to assume that populations living near railway stations will be more likely to use them (i.e., to be exposed) than populations living far from railway stations. In addition, and finally, studies have found that individuals who traveled to work using public transportation modes had bus and rail stops located closer to their workplace compared with respondents who commuted using private modes (e.g., car) (35). Thus, our assumption that those living near railway stations are more likely to travel by train is grounded on scientific evidence. This assumption, though plausible as urban populations tend to use the transportation means closest to their residence and chose their residential location based on proximity to public transportation (36), may not fully match individual-level public transportation utilization patterns. Ecological designs also limit our capacity to control for confounding, meaning that other factors (for which information was unavailable) rather than proximity to railway stations or socioeconomic deprivation may partially explain our findings (e.g., other modes of transportation, types of activities taking place in each area, population ethnic composition, etc.). Second, our study may be affected by the Modifiable Areal Unit Problem (37), which occurs when the number of spatial units (the scale) used to define the same area affects the study conclusions. If the geographical units are large, it is more likely that associations found at the aggregate level will diverge from the same associations found at the individual level leading to the so-called ecological fallacy (38). Although we used the smallest geographic units possible (smallest unit of epidemiological data dissemination), the use of parishes as the territorial unit of analysis could potentially “wash away” (gerrymander) differences in covariates and outcomes as they can hold substantial heterogeneity in terms of infection rates, socioeconomic profile and access to railway transportation. Consequently, due to this averaging effect, the observed associations may be underestimated, and/or we may have failed to identify significant associations and inequalities (39). A third issue, is the Uncertain Geographic Context Problem (40). Case data is available according to the parish of occurrence, but focusing only on occurrence location may introduce uncertainty in research results, because people may spend a considerable amount of time in other parishes and may acquire the disease in these locations (e.g., work, transportation, etc.) (41). Finally, case data only includes cases reported to the national surveillance system, which may not be sufficient to fully comprehend the true magnitude of the COVID-19 pandemic. Although the true number of undetected cases is still to be ascertained, in Europe, the ratio of the total estimated cases to the observed cases was found to be around 2.3 (42).

In conclusion, we found no consistent association between proximity to railway stations and SARS-CoV-2 infection rates in the most affected metropolitan area of the country, suggesting other factors, namely neighborhood socioeconomic deprivation, play a more prominent role in the epidemic dynamics. Nevertheless, our findings do not imply that safety measures in public transportation can be relaxed—proper surface cleaning and disinfecting, physical distancing, and mass mask use should continue to be promoted. To guide measures of epidemic control, individual-level studies, namely through the adoption of case-control designs, are recommended to better understand in which locations (e.g., work, school, and shopping) there is a higher risk of acquiring the infection.

## Data Availability Statement

The datasets presented in this article are not readily available because, spatial data can be fully provided upon request to the corresponding author. Patient data can be provided at municipality-level upon request to the corresponding author or can be downloaded *via* DGS website (https://www.dgs.pt/home.aspx?cpp=1). Requests to access the datasets should be directed to milton@med.up.pt.

## Ethics Statement

The studies involving human participants were reviewed and approved by Ethical approval was obtained from the Ethics Committee of the ISPUP, Portugal (approval number CE20153). Written informed consent from the participants' legal guardian/next of kin was not required to participate in this study in accordance with the national legislation and the institutional requirements.

## Author Contributions

MS: conceptualization, statistical analysis, interpretation of the results, and writing—review and editing. AR: geographical data processing, interpretation of the results, and writing—original. RL and TL: interpretation of the results and writing—review and editing. HB: conceptualization, supervision, interpretation of the results, and writing—review and editing. All authors contributed to the article and approved the submitted version.

## Conflict of Interest

The authors declare that the research was conducted in the absence of any commercial or financial relationships that could be construed as a potential conflict of interest.
